# Genetic Testing in Inherited Retinal Disease: Current Strategies and Future Directions

**DOI:** 10.3390/jpm16060288

**Published:** 2026-05-27

**Authors:** Sujin Kang, Byron L. Lam, Winston Lee, Audina M. Berrocal, Ninel Z. Gregori, Carlos E. Mendoza-Santiesteban, Jesse D. Sengillo

**Affiliations:** Department of Ophthalmology, Bascom Palmer Eye Institute, University of Miami Miller School of Medicine, 900 NW 17th Street, Miami, FL 33136, USA

**Keywords:** genetic testing, inherited retinal disease, precision medicine, next-generation sequencing, panel-based sequencing, whole-exome sequencing, whole-genome sequencing, artificial intelligence

## Abstract

Inherited retinal diseases (IRDs) are a major cause of visual impairment worldwide, marked by extensive genetic and phenotypic heterogeneity. Recent estimates from the U.S. suggest a prevalence of nearly 1 in 1000 individuals, reflecting both disease burden and improved diagnostic recognition. This review traces the shift from linkage analysis and Sanger sequencing to high-throughput next-generation sequencing, including panel-based, whole-exome, and whole-genome sequencing. Phenotype-driven testing strategies and standardized variant interpretation frameworks, such as the American College of Medical Genetics and Genomics guidelines, have substantially increased diagnostic yield. Copy number and structural variant detection, transcriptomics, and functional assays further help address unresolved cases. Nonetheless, barriers remain regarding cost, access, and the interpretation of variants of uncertain significance. Molecular confirmation has become essential for access to novel gene-directed therapies, exemplified by voretigene neparvovec for biallelic *RPE65* variants, and is often a prerequisite for clinical trial participation. The growing role of genetic testing highlights the need for multidisciplinary evaluation and standardized outcome measures. Emerging tools, including artificial intelligence-assisted variant prioritization, image-to-genotype modeling, and multi-omics analyses, bridge molecular diagnoses with clinical phenotypes, accelerating the transition to targeted therapies. Continued progress will depend on increased access, standardized analytical regulations, and the integration of emerging technologies into routine clinical care.

## 1. Introduction

Inherited retinal diseases (IRDs) represent a diverse group of genetic disorders that stand as a leading cause of blindness worldwide [1,2,3]. Recent U.S. data estimate a prevalence of 106 per 100,000 and an incidence of 15.5 per 100,000 [4], reflecting both improved recognition and advances in cellular and molecular diagnostics. Over the past two decades, genetic testing has evolved from linkage analysis and Sanger sequencing to next-generation sequencing (NGS), including panel-based sequencing, whole-exome sequencing (WES), and whole-genome sequencing (WGS) [5,6,7]. Although these approaches have improved diagnostic yield [8,9], many cases remain unsolved. This is often due to noncoding, splicing, and structural variants (SVs) that conventional panels or short-read WES (srWES) miss. WGS and functional splicing assays have helped address this limitation [6,8,10,11].

Despite these advances, significant clinical variability persists even among individuals sharing the same causal variant [12,13]. Variable expressivity, modifier loci, and regulatory or epigenetic influences likely modulate disease severity and progression beyond the primary mutation itself [14,15]. Addressing this discordance demands integrative approaches that align molecular findings with detailed clinical and imaging data, transcriptomic and proteomic analyses, and functional assays.

The clinical importance of precise genotyping has grown with the advent of gene-targeted therapies, exemplified by voretigene neparvovec (Luxturna^®^) for biallelic RPE65 variants, transforming molecular testing from a diagnostic tool to a therapeutic gateway. Emerging artificial intelligence (AI) and machine-learning (ML) tools can correlate genotype, multimodal imaging, and Online Mendelian Inheritance in Man (OMIM) data [16,17]. These approaches may improve understanding of disease mechanisms and refine individualized treatment strategies.

This review examines the evolution of genetic testing in IRDs and the ongoing challenge of achieving accurate molecular diagnoses. We also discuss how integrative genomics, functional validations, and AI-driven analyses may advance the field.

## 2. Current State of Genetic Testing in IRDs

### 2.1. The Next-Generation Sequencing Era

#### 2.1.1. Evolution of Genetic Testing and Diagnostic Yield

The molecular investigation of IRDs has undergone a fundamental transformation over the past three decades. Early efforts relied on linkage analysis in families to map disease loci, followed by Sanger sequencing of candidate genes, an approach that, while precise, was limited in scalability and cost-effectiveness given that more than 340 genes are currently implicated in IRDs (RetNet; https://retnet.org, accessed on 12 May 2026) [18]. Array-based genotyping enabled simultaneous screening of hundreds of known variants but could not detect novel or rare changes, limiting diagnostic sensitivity [19,20].

#### 2.1.2. Next-Generation Sequencing

The introduction of NGS in the early 2010s enabled massively parallel interrogation of multiple IRD genes in a single assay. Three principal strategies are now employed: targeted gene panels, WES, and WGS. Each differs in genomic coverage, cost, and capacity to detect noncoding and structural variants. The American Academy of Ophthalmology guidelines indicate that a causative mutation can be identified in 57–76% of patients with IRDs [21]. However, a 2023 meta-analysis by Britten-Jones and coworkers provides a more tempered perspective, reporting a pooled yield of 61.3% while highlighting extreme heterogeneity (I^2^ = 92%), which suggests that real-world clinical yields may be lower than those reported in specialized research cohorts. Meta-regression data indicate that diagnostic yields have plateaued since 2014, suggesting a performance ceiling for short-read sequencing. Outcomes also fluctuate significantly by phenotypic stratification, with yields dropping to 47.6% for conditions like familial exudative vitreoretinopathy. These results are further confounded by variability in sequencing pipelines, bioinformatic workflows for structural variants, and population-specific biases. Since mutation databases are disproportionately enriched with European and North American data, individuals from underrepresented ancestral backgrounds frequently face higher rates of variants of uncertain significance (VUSs). Furthermore, technical challenges in difficult-to-sequence regions, such as the *RPGR ORF15* exon, continue to impede sensitivity, illustrating the substantial impact of methodological and cohort heterogeneity on reported clinical outcomes [22]. Overall, reported diagnostic yields across IRD studies should be interpreted cautiously because study populations and criteria differ in relevant ways; some cohorts enroll general, clinically suspected IRD cases, whereas others evaluate highly selected cohorts at specialized centers with extensive phenotyping expertise. In addition, some studies require strict American College of Medical Genetics and Genomics (ACMG)-classified pathogenic or likely pathogenic variants, while others include candidate or phenotype-supported variants of uncertain significance. Together, these differences can significantly influence pretest probability and reported diagnostic performance independent of the sequencing technology itself.

#### 2.1.3. Panel-Based Sequencing

Targeted NGS panels remain one of the most widely used approaches for the molecular diagnosis of IRDs. Panel designs vary widely. Broad IRD panels capture most known IRD-associated genes (i.e., 269–409 genes across platforms) [23], whereas others restrict analysis to clinically selected subsets of genes. These narrower panels may be organized by disease category (e.g., RP, cone–rod dystrophy, or macular dystrophy) [23], by suspected inheritance pattern (e.g., autosomal dominant, autosomal recessive, or X-linked) [24], or by biologic pathway (e.g., phototransduction cascade genes, cilia-related genes, or pathways involved in retinal metabolism and structural integrity) [25,26,27,28]. Because panel content depends on how genes are selected and updated, performance and diagnostic scope can vary considerably across platforms.

Targeted panels typically achieve higher read depth across selected exons, making them especially effective for detecting single-nucleotide variants (SNVs) and small insertions/deletions in established disease genes. Panel testing can therefore be efficient, cost-effective, and clinically actionable when the phenotype is sufficiently specific to justify either a focused disease-category panel or a broader IRD panel. In routine practice, detailed phenotyping with multimodal imaging and electrophysiology, including optical coherence tomography (OCT), fundus autofluorescence (FAF), and electroretinography (ERG), helps refine the likely disease category, suggest inheritance pattern and guide panel selection [23].

A major strength of panel-based testing is its analytical performance within targeted regions. Earlier comparative work in IRD showed that targeted panel testing can provide more complete coverage of disease-relevant loci than WES, with higher sensitivity for variant detection in those genes because of denser and more uniform capture. Missing key exons can directly delay or prevent an accurate clinical diagnosis [29]. When targeted panels return negative results, it is typically because the causative gene was omitted from the panel design, or the exon-focused architecture missed pathogenic deep-intronic or regulatory variants. A well-known example is the recurrent deep-intronic *CEP290* variant associated with Leber congenital amaurosis, commonly reported in the literature as c.7726 + 1137A > G under one transcript nomenclature; exon-centered assays may miss this variant unless the panel explicitly queries the intronic site [30]. Taken together, these considerations support a pragmatic role for panel-based sequencing as a first-tier test in patients with a well-defined phenotype and strong prior suspicion for a recognized IRD gene set, particularly when expert clinical phenotyping is available with OCT, FAF, and ERG. When panel testing is negative or inconclusive, reflexive escalation to WES or WGS, with orthogonal copy-number variant (CNV) analysis and functional validation where indicated, is increasingly recommended.

#### 2.1.4. Whole-Exome Sequencing

WES captures the protein-coding regions of the genome (~1–2% of total DNA; ~20,000 genes) together with limited flanking intronic sequence at exon–intron boundaries enabling detection of exonic and canonical splice-site variants. While it offers a platform for novel gene discovery and iterative data reanalysis, reported diagnostic yields vary dramatically, from 24% in some cohorts [22] to over 71% in others [31], reflecting profound heterogeneity of patient populations and study methodology. These discrepancies are driven by differences in phenotype stratification, prior testing history (e.g., excluding common variants), and the specific sequencing pipelines utilized. The 2025 study by Esteve-Garcia and colleagues emphasizes the value of a stepwise approach, demonstrating that systematic reanalysis with updated virtual panels and functional validation can elevate yield from 59.6% to 67.6% by resolving previously elusive variants [32].

The clinical utility of WES is nevertheless constrained by technical and population-based factors. Coverage uniformity is often suboptimal in GC-rich regions or highly homologous sequences (e.g., *RP1* and *RP1L1*), leading to “blind spots” that obscure pathogenic variants [33,34]. Furthermore, similar to targeted panels, WES provides limited detection of deep-intronic, promoter, and complex SVs [20,34]. The broader genomic scope also inherently increases the bioinformatic burden, frequently generating VUS in genes unrelated to the patient’s phenotype [20,35]. These “incidental findings” can cause patient anxiety and prompt additional work-up unrelated to vision loss. Due to cost and time-intensive analysis, WES is typically positioned as a second-tier test following negative panel results, or as a first-tier option for atypical or syndromic presentations [21].

#### 2.1.5. Whole-Genome Sequencing

##### Short-Read Whole-Genome Sequencing

SrWGS interrogates the entire genome without targeted enrichment, avoiding capture-related bias and enabling more uniform coverage across coding and noncoding regions, allowing detection of pathogenic variants in intronic, regulatory, and intergenic regions that may be missed by panel sequencing or WES [8]. This facilitates identification of pathogenic noncoding variants, CNVs, SVs, and complex alleles that may be missed by exon-focused assays, including variants in IRD genes with known deep-intronic or structurally complex mechanisms such as *CEP290*, *ABCA4*, *USH2A*, and *RPGR*.

Reported diagnostic gains from srWGS should be interpreted cautiously because most studies enroll selected patients who remain unresolved after panel testing, array-based testing, or WES. In a paired comparison of 46 individuals with IRD, Ellingford and coworkers identified 14 clinically relevant variants missed by targeted NGS, including large deletions and noncoding variants, and confirmed a molecular diagnosis in 11 of 33 previously unresolved individuals; however, the increased diagnostic yield was also influenced by cohort selection, phenotype distribution, and population structure [8].

Diagnostic yield also depends on phenotypic stratification and analytic strategy. WGS may be particularly useful in patients with atypical or syndromic disease, monoallelic findings in recessive genes, suspected deep-intronic variants, or structural rearrangements. Conversely, its incremental value may be lower when a well-defined phenotype has already been assessed with a high-quality, frequently updated panel [8,36]. Large rare-disease sequencing programs, including the UK 100,000 Genome Project, have highlighted the value of genome sequencing for identifying pathogenic variants in IRD genes with known deep-intronic hotspots—*CEP290* (Leber congenital amaurosis), *ABCA4* (Stargardt disease), and *USH2A* (Usher syndrome and retinitis pigmentosa)—that elude exome-centric strategies [37].

Despite these advantages, srWGS retains limitations related to read length (~150 bp). Short reads can be difficult to align accurately in repetitive or duplicated genomic regions, limiting detection of complex SVs and variants in highly repetitive loci [38].

##### Long-Read Whole-Genome Sequencing

Long-read sequencing (LRS) technologies generate reads spanning thousands to tens of thousands of base pairs, enabling improved characterization of structural variation, repetitive regions, and haplotypes. Platforms such as PacBio HiFi and Oxford Nanopore allow direct phasing of variants across long genomic intervals and can resolve regions that are challenging for short-read sequencing [39].

These capabilities are particularly relevant in IRD genetics. LRS can accurately interrogate the repetitive *RPGR* ORF15 region, a major mutational hotspot in X-linked retinitis pigmentosa that is difficult to sequence using short-read methods [38]. LRS also facilitates the detection of large deletions, inversions, mobile-element insertions, and complex alleles, which may explain cases unresolved by conventional sequencing [39,40].

A major clinical benefit of LRS is direct allelic phasing, determining if two heterozygous variants in an autosomal recessive gene reside in *cis* or *trans*. Short-read platforms cannot resolve this distinction natively, often requiring parental segregation analysis or alternative transcript sequencing to establish a definitive molecular diagnosis [41]. PacBio HiFi sequencing achieves per-read accuracy exceeding 99.9%, while Oxford Nanopore sequencing offers the additional ability to detect native DNA methylation, providing potential insights into regulatory mechanisms [39]. Although LRS remains more expensive and less standardized in clinical pipelines than srWGS, it is increasingly being used as a third-tier diagnostic approach for IRD cases unresolved after panel, exome, or short-read genome sequencing, particularly when phasing of recessive variants is required [32].

LRS enables direct phasing of variants captured on the same read or within overlapping read chains (haplotype blocks). Variants separated by distances exceeding read or block lengths may remain unphased. Repetitive sequences, pseudogenes, and mapping ambiguity can also reduce phasing confidence [42]. LRS is also more costly than panel or WES and requires high molecular weight DNA [43], making it less flexible with respect to sample type; blood-derived DNA is generally preferred, whereas other options like saliva or buccal swabs may be suboptimal.

#### 2.1.6. Integrated CNV/SV Detection

CNVs and SVs account for an estimated 5–15% of pathogenic alleles in IRDs, depending on the gene and population [44]. CNVs are an important cause of unsolved IRDs, and studies focused on previously negative cases have shown that CNV analysis can materially improve diagnostic yield [45]. Integration of CNV/SV calling into NGS workflows has improved detection, with clinical adoption becoming widespread in the mid-to-late 2010s (approximately 2017–2018 onward) [46,47,48]. Orthogonal methods such as multiplex ligation-dependent probe amplification (MLPA) or qPCR may be used to confirm clinically significant CNVs detected by NGS pipelines. Panel and WES read-depth-based algorithms detect multi-exon deletions and duplications but have reduced sensitivity for single-exon events and balanced rearrangements [47]. WGS supports a broader range of detection methods—including, read-depth, split-read, discordant-pair, and assembly based—achieving higher sensitivity with breakpoint-level resolution [49].

Integrated CNV/SV analysis should be standard in IRD testing pipelines. Failure to screen for structural variants in genes harboring recurrent CNVs (e.g., *EYS*, *USH2A*, *PRPF31*) risks missing pathogenic alleles, especially when only a single heterozygous coding variant is identified in an autosomal recessive gene that correlates with the phenotype [45,49].

#### 2.1.7. Functional RNA/Minigene Assays

A growing number of IRD-associated variants exert pathogenic effects through aberrant pre-mRNA splicing. Deep-intronic variants that create cryptic splice sites and synonymous or missense variants disrupting exonic splicing enhancers are frequently classified as VUS by sequence-based criteria alone. Although not yet part of routine clinical diagnostics in most settings, functional RNA and minigene assays are increasingly used as complementary tools to resolve the pathogenicity of such variants.

Minigene assays involve cloning the variant-containing region into a splicing reporter construct and analyzing mRNA products by RT-PCR, directly visualizing aberrant splicing (exon skipping, intron retention, cryptic splice site activation). These assays have been instrumental in reclassifying VUS in major IRD genes such as *ABCA4*, *USH2A*, and *CEP290*, providing PS3-level functional evidence under ACMG criteria [50,51]. When patient-derived RNA is available (blood, fibroblasts, or retinal organoids), direct transcript analysis by RT-PCR or RNA sequencing (RNA-seq) captures the native splicing context, though limited expression of retinal genes in non-ocular tissues restricts clinical applicability [52]. Functional validation of splice variants is becoming increasingly important as antisense oligonucleotide therapies targeting splicing defects are under development [53].

##### Integration of Transcriptomic Evidence

RNA-seq can directly detect aberrant splicing, allelic expression imbalance, and expression outliers invisible to DNA-based sequencing. In unresolved IRD cases, transcriptomic analysis has identified causative splicing defects in an additional 10–15% of cases, particularly involving deep-intronic or synonymous variants [51,54]. Tissue source is a critical consideration. Patient-derived iPSC–retinal organoids offer disease-relevant transcriptomic context at significant cost [55]. Blood and fibroblast RNA-seq, while more accessible, may miss retina-specific splicing events, though several groups have shown that non-retinal tissues can reveal aberrant splicing for variants in ubiquitously expressed IRD genes [52]. Standardized protocols for RNA-seq-based diagnostic adjuncts are under development and expected to enter routine pipelines as evidence matures.

## 3. Practical Workflow

### 3.1. Test Selection Algorithm

**Figure 1:**
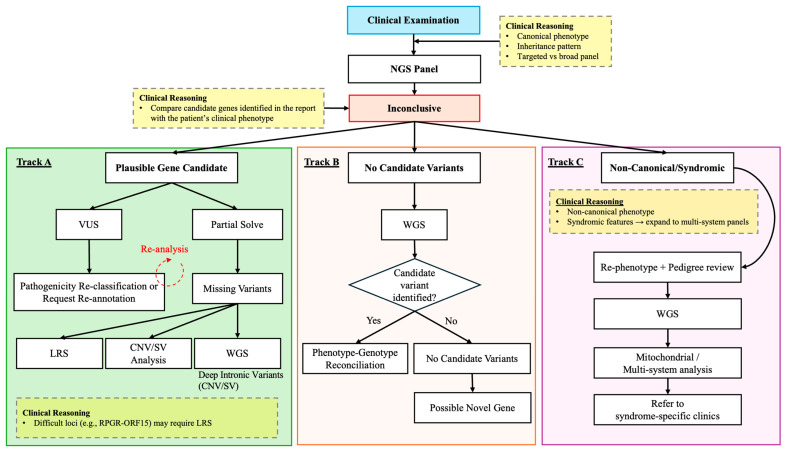
Proposed workflow for the clinical interpretation and escalation of genetic testing in patients with inherited retinal diseases following an inconclusive next-generation sequencing (NGS) panel result. Three major diagnostic tracks are illustrated based on the nature of the unresolved case. **Track A** includes cases with a plausible candidate gene or partial molecular diagnosis, prompting variant reinterpretation, re-analysis, or escalation to additional testing modalities such as long-read sequencing (LRS), whole-genome sequencing (WGS), or copy number/structural variant (CNV/SV) analysis to identify missing pathogenic variants (e.g., deep-intronic variants or difficult genomic loci such as *RPGR-ORF15*). **Track B** represents cases without clear candidate variants, in which WGS and phenotype–genotype reconciliation may support identification of novel candidate genes. **Track C** includes atypical, non-canonical, or syndromic presentations requiring re-phenotyping, pedigree review, mitochondrial or multi-system evaluation, and referral to syndrome-specific clinics. Yellow boxes highlight examples of clinical reasoning that may guide test selection and interpretation.

Genetic test selection for suspected IRD should follow a phenotype-driven, tiered approach (Figure 1). Thorough clinical phenotyping, including best-corrected visual acuity, OCT, FAF, and widefield fundus imaging, is an essential first step [21]. Electroretinography (full-field electroretinography [ffERG], multifocal electroretinography [mfERG]) may be a useful adjunct. A detailed family history with pedigree construction establishes the likely inheritance pattern, directly informing gene prioritization.

For well-defined phenotypes (e.g., typical rod–cone dystrophy with autosomal recessive inheritance), a targeted IRD gene panel is recommended as the first-tier test [21,32]. If panel testing does not identify two pathogenic alleles in a recessive gene or a single dominant/X-linked allele, WES with CNV analysis is the next step.

For pathognomonic phenotypes, such as X-linked retinoschisis, Stargardt, or Best disease, a single-gene testing can be considered. For atypical phenotypes, syndromic features, or prior negative testing, WGS with integrated SV detection can be considered a first-line approach.

When only a single heterozygous pathogenic variant is found in an autosomal recessive gene that correlates with the phenotype, targeted CNV analysis or investigation of noncoding/splice-altering variants using functional assays or RNA-seq may be considered for that gene, although some of these approaches are generally limited to specialized laboratories [37,54].

#### 3.1.1. Variant Interpretation and ACMG Guidelines

Variant interpretation in IRD diagnostics follows the ACMG/Association for Molecular Pathology (AMP) five-tier classification system: pathogenic, likely pathogenic, VUS, likely benign, and benign [56]. This framework integrates multiple evidence categories, including population frequency, computational predictions, functional data, segregation evidence, and prior clinical observations [23,56].

To address disease-specific considerations, ClinGen expert panels have developed gene- and disease-specific refinements to ACMG criteria [23]. For IRDs, the ClinGen Retinal Disorders Expert Panel has published curated rule specifications for several genes, improving consistency and reducing inter-laboratory variability in variant classification [57]. These tailored approaches are necessary to capture the genetic complexity within distinct groups, thereby improving diagnostic accuracy.

In clinical workflows, sequencing data undergo bioinformatic processing including read alignment, variant calling, and annotation [58]. Variants are then filtered based on allele frequency in population databases (e.g., Genome Aggregation Database [gnomAD]), predicted functional impact using computational tools (such as Combined Annotation Dependent Depletion [CADD], Rare Exome Variant Ensemble Learner [REVEL], and SpliceAI), and established gene–disease associations documented in resources including OMIM, RetNet, and ClinVar [58,59].

Variants that pass these filtering steps undergo manual curation, integrating genotype data with the patient’s phenotype, inheritance pattern, family segregation when available, and published evidence [23,58]. This multidisciplinary evaluation informs final ACMG classification and assessment of clinical relevance [60,61].

#### 3.1.2. Online Tools for VUS Predictability Assessment

Clinical laboratories primarily interpret VUSs using standardized frameworks that integrate population databases, disease-specific repositories, and in silico prediction tools. Publicly available resources such as ClinVar, Leiden Open Variation Database (LOVD), and gnomAD aggregate variant classifications, locus-specific data, and population allele frequencies that underpin laboratory assessments [62,63,64]. In addition, computational predictors including CADD, REVEL, SpliceAI, and AlphaMissense are routinely incorporated into laboratory pipelines [65,66,67,68].

For clinicians, these tools do not replace laboratory interpretation but provide transparency and a framework for contextual evaluation. Their utility lies in correlating genetic findings with detailed phenotypic data, assessing gene–disease relevance, and identifying situations where additional evidence, such as segregation analysis or functional studies, may support variant reclassification.

#### 3.1.3. VUS Reclassification: Practical Steps for Clinicians

**Figure 2:**
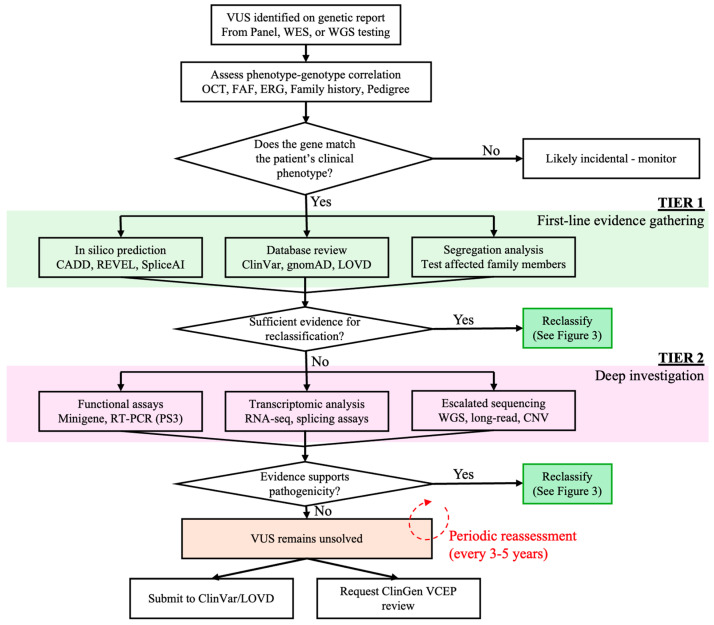
Suggested workflow for the interpretation and reclassification of variants of uncertain significance (VUS) identified through panel testing, whole-exome sequencing (WES), or whole-genome sequencing (WGS). Initial assessment includes evaluation of phenotype–genotype correlation using multimodal retinal imaging, electrophysiology, family history, and inheritance pattern. **Tier 1** outlines first-line evidence-gathering approaches including in silico prediction tools, population and disease databases, and segregation analysis in affected family members. If evidence remains insufficient for reclassification, **Tier 2** includes more extensive investigation through functional assays, transcriptomic analysis, and escalated sequencing approaches such as WGS, long-read sequencing, or CNV analysis. Cases that remain unresolved may undergo periodic reassessment as new evidence and genomic resources become available.

**Figure 3:**
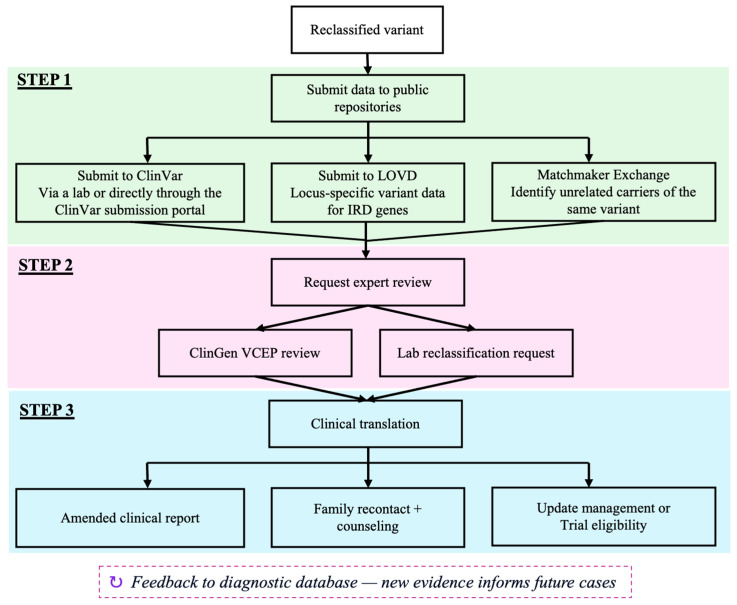
Proposed framework for the clinical reclassification and dissemination of reinterpreted IRD variants. **Step 1** involves dissemination of updated variant data to public genomic repositories and matchmaking platforms, including ClinVar, LOVD, and Matchmaker Exchange, to facilitate aggregation of supporting evidence and identification of unrelated carriers with similar phenotypes. **Step 2** includes expert review through ClinGen Variant Curation Expert Panels (VCEPs) or direct laboratory reassessment requests. **Step 3** focuses on the clinical translation of reclassified variants through amended diagnostic reports, patient recontact and counseling, and potential modification of clinical management or trial eligibility. The feedback loop shown at the bottom of the figure emphasizes how continuous data sharing and reinterpretation may improve future diagnostic accuracy in IRDs.

VUS classification is not permanent; reclassification occurs as evidence accumulates (Figure 2). Clinicians can contribute by submitting clinical and segregation data to ClinVar through the testing laboratory or directly via the ClinVar submission portal (Figure 3) [57,69]. Laboratories are receptive to reclassification requests when provided with additional segregation data, functional studies, or de novo occurrence evidence.

Clinicians can also request formal variant review of phenotypic–genotypic correlations from ClinGen Variant Curation Expert Panels (VCEPs) for retinal disease, which apply gene-specific ACMG specifications. Submitting cases to variant-sharing platforms (LOVD, Matchmaker Exchange) can identify additional unrelated carriers, strengthening pathogenicity evidence [62,70].

#### 3.1.4. Considerations for Repeat Genetic Testing

Repeat testing can be considered when the initial test was performed more than three to five years prior, as panel content, bioinformatic pipelines, and classification criteria evolve. If the initial test was a targeted panel, escalation to WES or WGS may be appropriate rather than repeating the same panel. If WES was the initial test, WGS with integrated SV and noncoding variant analysis offers the greatest incremental yield [58].

Data reanalysis, which applies updated pipelines and databases to existing sequencing data, is a cost-effective alternative that yields new diagnoses in 10–15% of previously unresolved IRD cases [58,71]. Clinicians should consider periodic reanalysis before ordering new tests.

#### 3.1.5. Real-World Barriers to Advanced Genomic Technologies

Insurance coverage for IRD genetic testing varies widely by payer, region, and testing modality. In the United States, voretigene neparvovec (Luxturna^®^) approval has strengthened the case for coverage as a therapeutic prerequisite, and commercial insurers and Medicare may cover targeted panel testing for clinically diagnosed IRD. However, approval for WES, WGS, RNA-seq, long-read sequencing, functional validation, and repeat analysis remains rare and inconsistent.

Beyond insurance, access is limited by institutional resources. Many non-specialized centers lack the dedicated genetic counseling, bioinformatic pipelines, and multidisciplinary teams required to interpret complex or noncoding variants [72]. Laboratory standardization also remains incomplete, with differences in gene content, sequencing platforms, coverage thresholds, CNV/SV pipelines, splice-prediction tools, ACMG/AMP application, and VUS reporting [37].

Underrepresentation of diverse populations in variant databases increases VUS rates and diagnostic uncertainty for patients of non-European ancestry, compounding diagnostic inequities. Programs such as the Foundation Fighting Blindness My Retina Tracker^®^ and no-cost testing programs from diagnostic laboratories and pharmaceutical companies have sought to mitigate these barriers, but gaps persist [72,73]. Access to testing also varies globally, with many low- and middle-income countries lacking specialized IRD genetic testing infrastructure. Therefore, advanced genomic technologies should be implemented through phenotype-driven, tiered workflows supported by data sharing, ancestry-diverse reference databases, reimbursement reform, and clearer laboratory standards.

## 4. Genetic Testing as the Gateway to Therapy

### Clinical Trial Eligibility and Outcome Standardization

The 2017 approval of voretigene neparvovec (Luxturna^®^) for biallelic *RPE65*-associated retinal dystrophy established that precise molecular diagnosis is a prerequisite for therapeutic access [74,75]. Patients must carry two pathogenic or likely pathogenic *RPE65* variants confirmed by a CLIA-certified laboratory to be eligible for therapy. This model has defined the paradigm for subsequent IRD gene therapy trials.

The IRD gene therapy pipeline now includes trials targeting *RPGR*, *CHM*, *CNGA3*, *CNGB3*, *RS1*, *RHO*, *ABCA4*, and *USH2A*, among others. Each trial defines molecular eligibility criteria including variant type, location, zygosity, and sometimes functional parameters (residual acuity, retinal structure on OCT). Accurate genetic testing is essential for both diagnosis and trial eligibility [76,77]. Emerging gene-editing strategies, including CRISPR-based approaches, are also under investigation for selected IRD genes [78].

The field has moved toward harmonized outcome measures including the multi-luminance mobility test (MLMT), full-field stimulus testing (FST), microperimetry, OCT- and autofluorescence-derived metrics (e.g., ellipsoid zone area), and patient-reported outcomes [79,80,81]. The Foundation Fighting Blindness Clinical Consortium and International Society for Clinical Electrophysiology of Vision have contributed standardized protocols that facilitate cross-trial comparisons [82,83].

## 5. Emerging Future Directions

### AI/ML and Image-to-Genotype Modeling

Deep-learning algorithms trained on multimodal retinal imaging, including OCT, FAF, adaptive optics scanning laser ophthalmoscopy (AO-SLO), and widefield fundus photography have demonstrated the capacity to identify imaging features that correlate with specific IRD genotypes, sometimes approaching expert-level diagnostic accuracy [84,85,86,87,88].

Image-to-genotype modeling is a particularly promising frontier, but it is not ready to replace molecular genetic testing or expert clinical interpretation. Convolutional neural networks and vision transformers can predict genetic diagnoses directly from retinal images. In specific research settings, these models have successfully distinguished *ABCA4*-associated Stargardt disease from other macular dystrophies and predicted candidate genes with high accuracy [89]. Nevertheless, such models are vulnerable to confounding by referral patterns, ancestry-related genetic architecture, phenotypic overlap among IRDs, variable disease stage, and differences in image acquisition or preprocessing. In addition, most studies are trained on small, single-center, or retrospective datasets [86,90,91]. These results suggest that limited sample sizes for rare genotypes may reduce generalizability and increase the risk of model overfitting.

Longitudinal imaging datasets linked with genotype and clinical outcomes may eventually support models that estimate rates of photoreceptor degeneration, visual field loss, or treatment-relevant progression endpoints. At present, however, major barriers remain [17,92]. These challenges are particularly pronounced in IRDs because individual diseases and genotypes are rare, while a large proportion of patients remain genetically unresolved and therefore cannot be reliably incorporated into genotype-driven training models. Furthermore, the use of multiple imaging platforms introduces additional layers of variability that may impair reproducibility and model generalizability. Many current studies are also derived from highly selected tertiary referral cohorts enriched for canonical phenotypes and frequently include repeated images from the same patients or eyes [73,93], potentially inflating apparent model performance. Consequently, these AI/ML platforms should be viewed as adjunctive research tools to support diagnosis and trial design rather than stand-alone clinical diagnostic systems.

## 6. Conclusions

Genetic testing for IRDs has rapidly evolved with NGS technologies, enabling molecular diagnoses in the majority of well-phenotyped patients. Concurrently, the advent of gene-targeted therapies has elevated molecular diagnosis from an academic exercise to a therapeutic necessity, demanding comprehensive and accessible genetic testing for all IRD patients.

Looking ahead, AI/ML approaches promise to accelerate genotype–phenotype correlation and enable image-based diagnostic triage. Realizing this potential will require diverse well-curated datasets, equitable access to advanced testing, and collaborative frameworks spanning institutions and borders.

## Data Availability

Not applicable.

## Abbreviations

ACMG	American College of Medical Genetics and Genomics
AI	artificial intelligence
AMP	Association for Molecular Pathology
AO-SLO	adaptive optics scanning laser ophthalmoscopy
AUC	area under the curve
CADD	Combined Annotation Dependent Depletion
CLIA	Clinical Laboratory Improvement Amendments
CMA	chromosomal microarray analysis
CNN	convolutional neural network
CNV	copy-number variant
ERG	electroretinography
FAF	fundus autofluorescence
ffERG	full-field electroretinography
FST	full-field stimulus testing
gnomAD	Genome Aggregation Database
iPSC	induced pluripotent stem cell
IRD	inherited retinal disease
LLM	large language model
LOVD	Leiden Open Variation Database
LRS	Long-Read Sequencing
mfERG	multifocal electroretinography
ML	machine learning
MLMT	multi-luminance mobility test
MLPA	multiplex ligation-dependent probe amplification
NGS	next-generation sequencing
NLP	natural language processing
OCT	optical coherence tomography
OMIM	Online Mendelian Inheritance in Man
ONT	Oxford Nanopore Technologies
qPCR	quantitative polymerase chain reaction
REVEL	Rare Exome Variant Ensemble Learner
RNA-seq	RNA sequencing
RP	retinitis pigmentosa
SD-OCT	spectral-domain optical coherence tomography
SNV	single-nucleotide variant
srWGS	short-read whole-genome sequencing
SS-OCT	swept-source optical coherence tomography
SV	structural variant
VUS	variant of uncertain significance
WES	whole-exome sequencing
WGS	whole-genome sequencing
